# Supplementary material for: Kramers nodal lines in intercalated TaS2 superconductors
Source: Nat Commun. 2025 May 29;16:4984. doi: 10.1038/s41467-025-60020-z (PMC12122674; doi:10.1038/s41467-025-60020-z)
Supplement: Supplementary file 1 — Supplementary Information [file 41467_2025_60020_MOESM1_ESM.pdf]

Supplementary Information for "Kramers nodal lines in intercalated TaS_2 superconductors"

Yichen Zhang[1,†], Yuxiang Gao[1,†], Aki Pulkkinen[2,†], Xingyao Guo[3], Jianwei Huang[1], Yucheng Guo[1], Ziqin Yue[1,4], Ji Seop Oh[1,5], Alex Moon[6], Mohamed Oudah[7], Xue-Jian Gao[3], Alberto Marmodoro[2], Alexei Fedorov[8], Sung-Kwan Mo[8], Makoto Hashimoto[9], Donghui Lu[9], Anil Rajapitamahuni[10], Elio Vescovo[10], Junichiro Kono[1,11,12,13,14], Alannah M. Hallas[7,15,16], Robert J. Birgeneau[5,17], Luis Balicas[6], Ján Minár[2], Pavan Hosur[18], Kam Tuen Law[3], Emilia Morosan[1,11,14,*], Ming Yi[1,11,14,*]

[1]*Department of Physics and Astronomy, Rice University, Houston, Texas 77005, USA*

[2]*New Technologies Research Center, University of West Bohemia, Plzen 301 00, Czech Republic*

[3]*Department of Physics, Hong Kong University of Science and Technology, Clear Water Bay, Hong Kong, China*

[4]*Applied Physics Graduate Program, Smalley-Curl Institute, Rice University, Houston, Texas 77005, USA*

[5]*Department of Physics, University of California, Berkeley, California 94720, USA*

[6]*National High Magnetic Field Laboratory, Tallahassee, Florida 32310, USA*

[7]*Stewart Blusson Quantum Matter Institute, University of British Columbia, Vancouver, British Columbia V6T 1Z4, Canada*

[8]*Advanced Light Source, Lawrence Berkeley National Laboratory, Berkeley, California 94720, USA*

[9]*Stanford Synchrotron Radiation Lightsource, SLAC National Accelerator Laboratory, 2575 Sand Hill Road, Menlo Park, California 94025, USA*

[†]These authors contributed equally: Yichen Zhang, Yuxiang Gao, Aki Pulkkinen

[*]To whom correspondence should be addressed: emorosan@rice.edu, mingyi@rice.edu

[10]*National Synchrotron Light Source II, Brookhaven National Lab, Upton, NY 11973, USA*

[11]*Rice Center for Quantum Materials, Rice University, Houston, Texas 77005, USA*

[12]*Department of Electrical and Computer Engineering, Rice University, Houston, Texas 77005, USA*

[13]*Department of Materials Science and NanoEngineering, Rice University, Houston, Texas 77005, USA*

[14]*Smalley-Curl Institute, Rice University, Houston, Texas 77005, USA*

[15]*Department of Physics & Astronomy, University of British Columbia, Vancouver, British Columbia V6T 1Z1, Canada*

[16]*Canadian Institute for Advanced Research, Toronto, Ontario M5G 1M1, Canada*

[17]*Materials Science Division, Lawrence Berkeley National Laboratory, Berkeley, California 94720, USA*

[18]*Department of Physics and Texas Center for Superconductivity, University of Houston, Houston, Texas 77204, USA*

Contents

Supplementary Note 1: Rietveld refinement on powder x-ray diffrraction spectrum of In_xTaS_2 (x = 1/2 and 1) **5**

Supplementary Note 2: Theoretical Green's function-based calculations on $In_{0.67}TaS_2$ **7**

Supplementary Note 3: Spatial homogeneity and the non-stoichiometry of $In_{1/2}TaS_2$ **9**

Supplementary Note 4: Photon-energy-dependence of the angle-resolved photoemission data of In_xTaS_2 **9**

Supplementary Note 5: Angle-resolved photoemission termination dependence of $InTaS_2$ and $In_{1/2}TaS_2$ **16**

Supplementary Note 6: Kramers nodal lines in the isostructural $PbTaSe_2$ **20**

Supplementary Note 7: First-principles calculations and quantum oscillations on $InTaS_2$ **22**

Supplementary Note 8: Chiral Majorana modes and anomalous Hall effect from an ideal Kramers nodal line **27**

Supplementary References **34**

Supplementary Note 1: Rietveld refinement on powder x-ray diffraction spectrum of In_xTaS_2 (x=1/2 and 1)

Two types of structures (main text Fig. 1(b)) are reported on In_xTaS_2 depending on the In position in the unit cell in the inorganic crystal structure database (ICSD). The relative peak intensity of the calculated x-ray diffraction (XRD) pattern of the two structures is quite different even if the indium occupation is set to be the same. Therefore, we performed Rietveld refinement on powder X-ray diffraction to determine the exact structure of In_xTaS_2 (x = 1/2 and 1). The powder XRD spectrum of both compositions (x = 1/2 and 1) is fitted with both structures. The Rietveld refinement with $InTaS_2$ structure (right of main text Fig. 1(b), In on top of S) on the powder XRD spectrum of $InTaS_2$ yields a χ^2 = 5.45 (Supplementary Fig. 1(a)), while the refinement with $In_{1/2}TaS_2$ structure (left of main text Fig. 1(b), In on top of Ta) yields a χ^2 = 42.7 (Supplementary Fig. 1(b)). This suggests that $InTaS_2$ crystallizes in the structure with In on top of S as in the left of main text Fig.1(b). On the contrary, the Rietveld refinement with $InTaS_2$ structure on the powder XRD spectrum of $In_{1/2}TaS_2$ yields a χ^2 = 22.6 (Supplementary Fig. 1(c)), while the refinement with $In_{1/2}TaS_2$ structure yields a χ^2 = 8.68 (Supplementary Fig. 1(d)). This suggests that $In_{1/2}TaS_2$ crystallizes in the structure of In on top of Ta (right of main text Fig. 1(b)). Despite the difference in the crystal structure of $InTaS_2$ and $In_{1/2}TaS_2$, the two structures have the same space group symmetry ($P\bar{6}m2$) and the time reversal invariant momenta. The Kramers nodal lines are expected to be at the same locations in the Brillouin zone. The lattice parameters for x = 1/2 and 1 are also refined from the Rietveld refinement: a = 3.3102 Å and c = 7.9372 Å for x = 1/2, and a = 3.2854 Å and c = 8.7073 Å for x = 1.

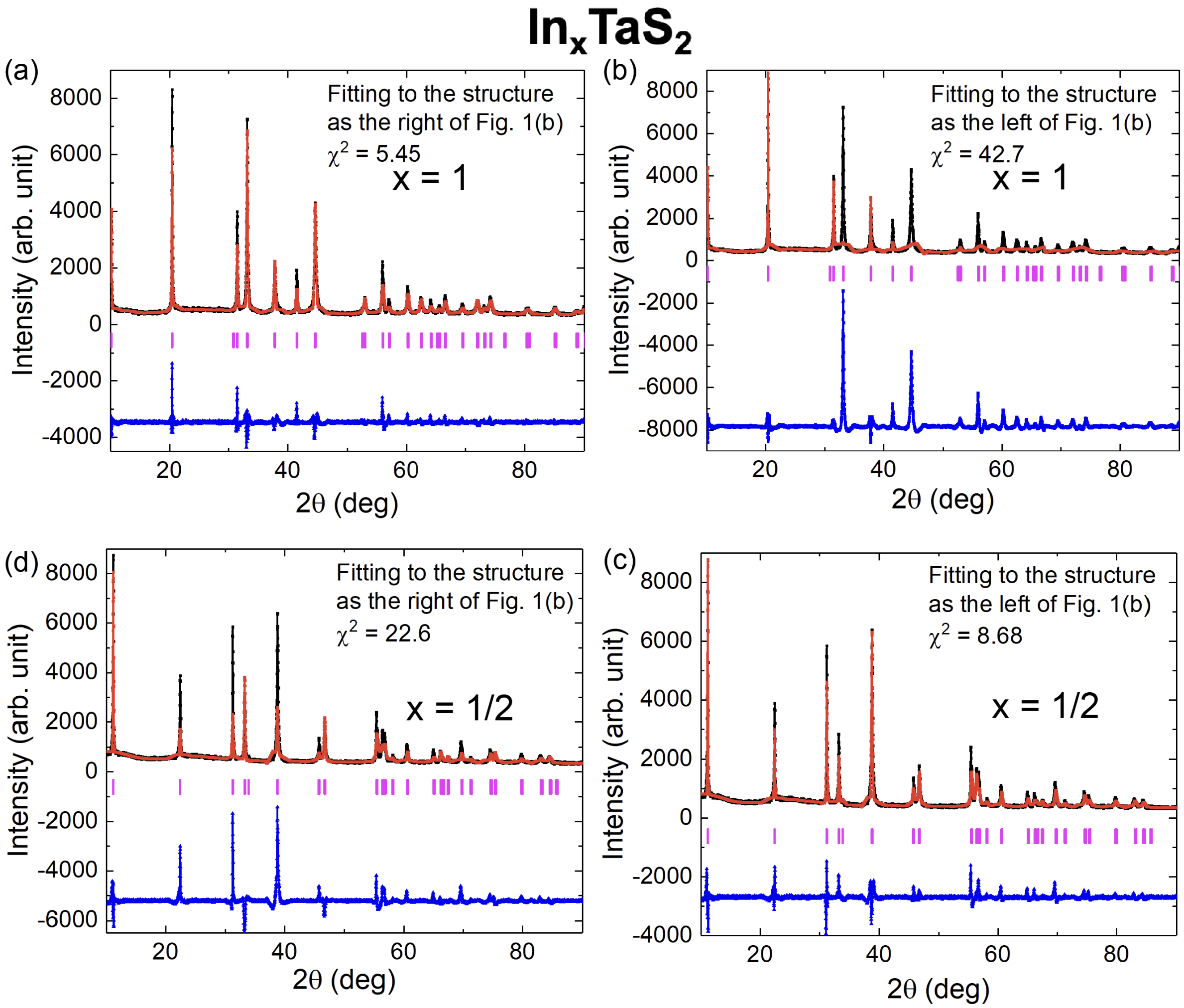

Supplementary Figure 1: Rietveld refinement on powder XRD spectrum of In_xTaS_2 (x = 1/2 and 1). Black lines: experimental data. Red lines: calculation result. Magenta symbols: Bragg peak positions. Blue lines: the difference between experiments and calculation. (a-b) XRD pattern of $InTaS_2$ with refinement on $InTaS_2$ structure (a) and $In_{1/2}TaS_2$ structure (b). (c-d) XRD pattern of $In_{1/2}TaS_2$ with refinement on $InTaS_2$ structure (c) and $In_{1/2}TaS_2$ structure (d).

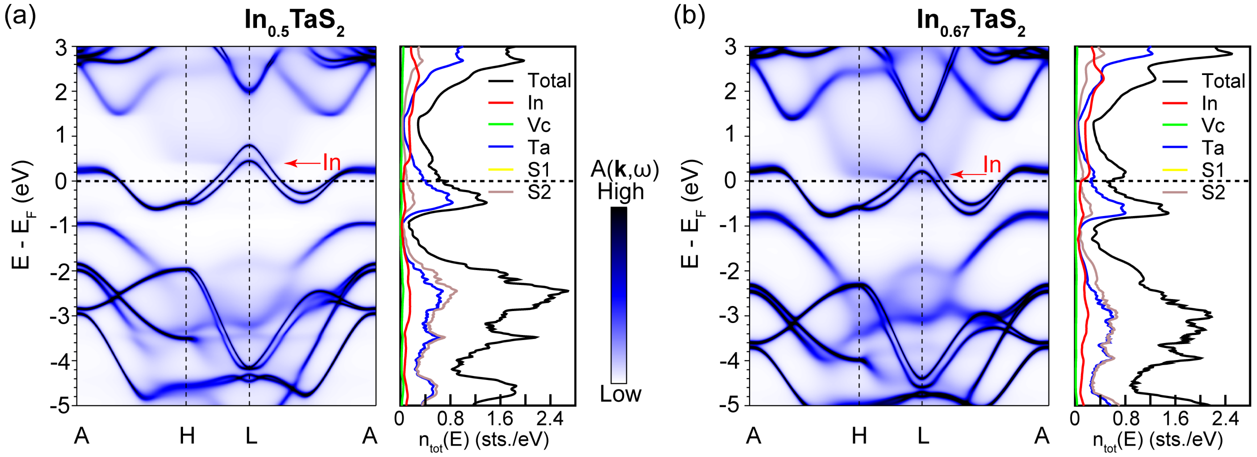

Supplementary Figure 2: Comparison of the calculated bulk Bloch spectral function and site-resolved density of states (DOS) between $In_{0.5}TaS_2$ (a) and $In_{0.67}TaS_2$ (b). Red arrows indicate the incipient indium spectral intensity. Vc: vacancy on the indium site. S1 and S2 stand for the two sulfur atoms in the unit cell. They share the same band dispersions and DOS in the final results, but were treated as individual sites in the presented full-potential fully-relativistic Korringa-Kohn-Rostoker Green's function-based calculations.

Supplementary Note 2: Theoretical Green's function-based calculations on $In_{0.67}TaS_2$

Based on the fact that the Korringa-Kohn-Rostoker Green's function (KKR-GF) calculations provide excellent descriptions on the electronic properties of $In_{0.5}TaS_2$, as demonstrated in main text Fig. 2, we compare, in this section, the calculated electronic band dispersions and density of states (DOS) of $In_{0.67}TaS_2$ with those of $In_{0.5}TaS_2$, and provide insights on the T_C enhancement in $In_{0.67}TaS_2$.

As shown by the red arrow in Supplementary Fig. 2(a), the In spectral intensities in $In_{0.5}TaS_2$

near A-H-L-A directions are weak near the Fermi level E_{F} until E = E_{F} + 200 meV. This is also confirmed by the k-integrated DOS attached on the right for the same binding energy range. The Ta d orbitals dominate near E_{F}, while the In DOS (red curve) shows a step-like drop at around E_{F} + 200 meV. On the other hand, notably for $In_{0.67}TaS_2$ in Supplementary Fig. 2(b), the In site shows stronger incipient spectral intensity at the Fermi level, and its DOS becomes comparable with Ta slightly above E_{F}. Therefore, the total DOS develops a two-peak structure around E_{F}, with a dip at the Fermi level due to the sharp drop of In DOS (red curve) from the $5p$ orbitals. For the superconductivity in the $1H$-TaS_2 layers, it is dominated by the Ta d orbitals. Since In atoms are located above the Ta atoms in $In_{0.5}TaS_2$ and $In_{0.67}TaS_2$, this observation reveals that more In orbitals will participate in the superconducting state of $In_{0.67}TaS_2$, which likely contributes to the higher T_{C} and the interlayer coherence. This is consistent with the clear T_{C} drop in $InTaS_2$, as the In atoms move on top of the S atoms. In addition, an electron-doping trend occurs from $In_{0.5}TaS_2$ to $In_{0.67}TaS_2$. Recent theory study on doping dependence of monolayer $1H$-TaS_2 reveals that under the consideration of spin-orbit coupling, electron doping weakens the CDW instability, yields strengthened electron-phonon coupling, and enhances the superconducting T_{C} [1]. Our experimental observations and fully relativistic calculation results are consistent with such theory indication. However, a detailed study on understanding the superconductivity in In_xTaS_2 treating In disorder, its doping effect, and electron-phonon coupling on the same footing requires further efforts.

Supplementary Note 3: Spatial homogeneity and the non-stoichiometry of $In_{1/2}TaS_2$

In order to confirm the real-space homogeneity and the chemical composition of the In_xTaS_2 single crystals, we exfoliated from the same piece of the sample that was used for angle-resolved photoemission spectroscopy (ARPES) and spin-resolved ARPES measurements presented in the main text Figs. 2 and 3 and performed scanning electron microscopy with energy dispersive x-ray spectroscopy (SEM-EDX) experiments. The results in Supplementary Fig. 3(a-c) demonstrate that the presence of In, Ta, and S is homogeneous across the entire scanned region. We also performed one-dimensional EDX scans at different regions of the same sample, which showed a consistent atomic composition of In:Ta:S $\approx$ 0.53:0.99:2, close to the $In_{1/2}TaS_2$ convention adopted. The result is presented in Supplementary Fig. 3(d).

Supplementary Note 4: Photon-energy-dependence of the angle-resolved photoemission data of In_xTaS_2

Both $In_{1/2}TaS_2$ and $InTaS_2$ are quasi-two-dimensional (2D) materials with the building blocks of In-layer and TaS_2-layer. One would expect that the two materials exhibit weak photon-energy-dependence of the band dispersions in the vacuum ultraviolet (VUV) regime of incident photons, complicated by kinetic-energy-dependent matrix element effects that vary with the photon energy. To clarify their photon-energy-dependence and have a rough estimate of the k_z positions of the acquired data, we present in Supplementary Fig. 4 and Supplementary Fig. 5 the ARPES $h\nu$-

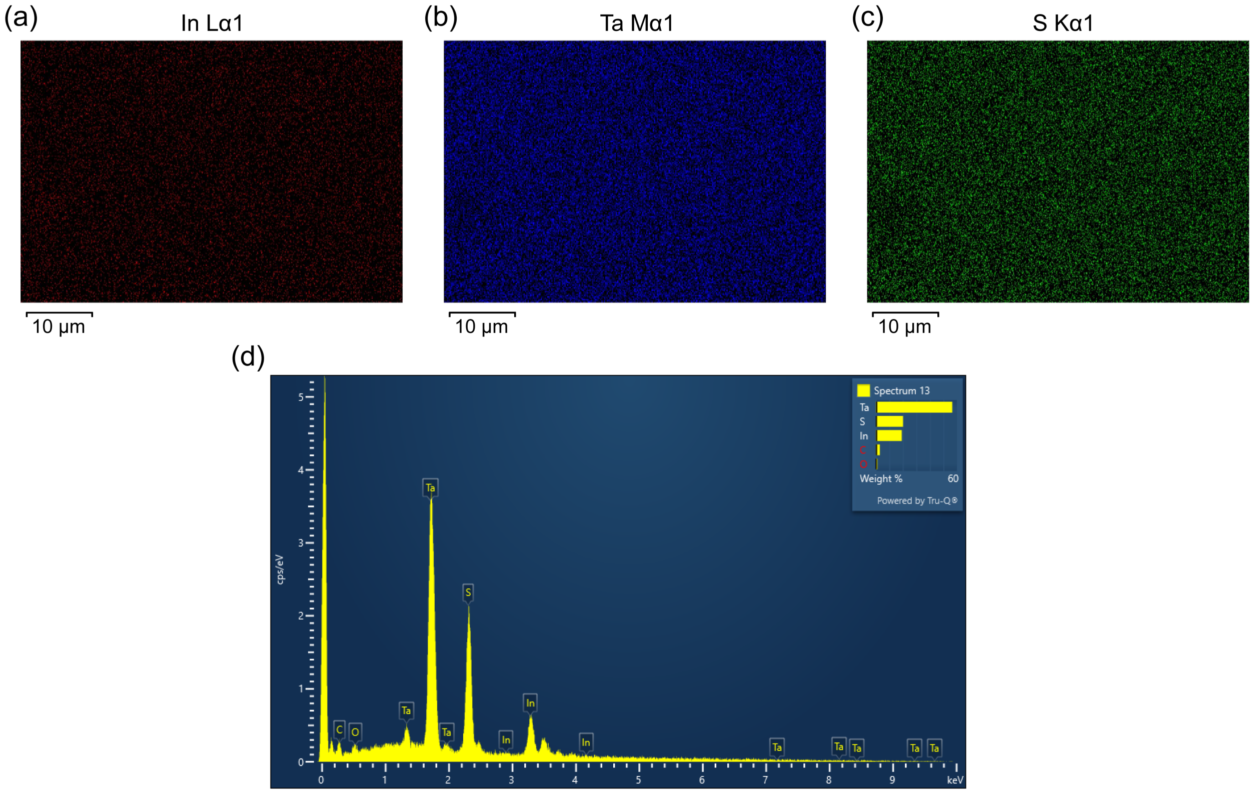

Supplementary Figure 3: Real-space scanning electron microscopy with energy dispersive x-ray spectroscopy (SEM-EDX) results of $In_{1/2}TaS_2$ at room temperature. (a-c) Spatial scan for the x-ray emitted by electrons returned to the L shell of In, the M shell of Ta, and the K shell of S, respectively. (d) Area-averaged EDX spectrum to determine the elemental composition of the nominal $In_{1/2}TaS_2$, yielding $In_{0.53}Ta_{0.99}S_2$.

dependence of $In_{1/2}TaS_2$ and $InTaS_2$, respectively. We note that one can estimate the k_z resolution of VUV-ARPES using the inelastic mean free path of photoelectrons λ_{IMFP}: $\Delta k_z \approx \hbar/\lambda_{IMFP}$[2], where $\hbar$ is the reduced Planck constant. Typically for VUV photons, Δk_z is around the order of 0.1 Å^{-1}. Therefore, we emphasize that our results only provide a rough estimate of the k_z dispersions in these layered materials.

As shown in Supplementary Figs. 4(a) and (b), the isolated Kramers nodal line (KNL) band along $\bar{\Gamma}-\bar{M}$ in In_xTaS_2 shows two straight line-like features along k_z near the Fermi level, indicating that the Fermi surface is highly 2D, consistent with the 3D voxel-style Fermi surface topology of $In_{1/2}TaS_2$ calculated in the main text Fig. 1(e). The k_z dispersionless feature is also facilitated by the surface-sensitivity of VUV APRES. We note that the $In_{1/2}TaS_2$ samples have doping variations even within the same batch of synthesis. Therefore, the x of In concentration could vary near 0.5. From the hole-doping trend of the band positions in Supplementary Fig. 4, in comparison with the main text Figs. 2 and 3, we speculate that this sample has a slightly lower concentration of In, but also close to x = 0.5. Furthermore, upon examining the E-k_x-k_z ARPES data at deeper binding energies of 1 and 1.2 eV, as shown in Supplementary Figs. 4(c) and (d), more k_z dispersive features show up. To determine the inner potential for the photon energy $h\nu$ to k_z conversion, we examine the $E - k_z$ band dispersions in Supplementary Fig. 4(e), taken from the vertical (V) cut in Supplementary Fig. 4(c), in comparison with the Korringa-Kohn-Rostoker density functional theory (KKR-DFT) results along $\Gamma - A$. It is clear in the DFT prediction that a k_z-dispersive band reaches its band-top around 1 eV binding energy at A ($k_z = \pi$), accompanied by the non-k_z-dispersive bands at around E - E_F = -2 eV. Therefore, we interpreted our photon-energy-dependent ARPES data

with an inner potential choice of 13 eV, so that the k_z dispersive intensities at high photon energies (higher k_z) in Supplementary Fig. 4(e) are centered around the Γ near k_z = 5.5 Å^{-1} and match with the characteristic of band-top appearing at A. Such Γ-symmetric feature is also denoted by the two red horizontal arrows in Supplementary Fig. 4(d) for the $k_x - k_z$ constant energy contour at E - E_{F} = -1.2 eV. Notice that the higher the photon energy is, the better the k_z resolution is. Therefore, data at lower photon energy (lower k_z) can be poorly resolved, while modulated by photoemission matrix element effects. Despite such complications, we note that the k_z-dispersive band still reaches its top for another A near k_z = 4.3 Å^{-1} (see Supplementary Fig. 4(e)), under the 13 eV inner potential. Further, we point out that the non-k_z-dispersive bands predicted by DFT at around 2 eV binding energy along $\Gamma - A$ are enhanced for the ARPES data between k_z = 4.4 and 5.1 Å^{-1} in Supplementary Fig. 4(e), likely due to the kinetic-energy-dependent photoemission matrix element affected by the final-state effects. In Supplementary Figs. 4(f) and (g), we subsequently showcase the $E - k_x$ band dispersions corresponding to the 78 and 96 eV indicated in Supplementary Fig. 4(c), respectively, while half of the ARPES data are replaced by the DFT calculations at the correspondingly constant k_z for an in-depth comparison. Importantly, the red dashed boxes in Supplementary Figs. 4(f) and (g) emphasize the appearance of the A band-top near 1 eV binding energy at 96 eV (near $k_z = \pi$) but its disappearance at 78 eV (near $k_z = 0$). Therefore, the 75 eV photon energy employed in the main text Figs. 2 and 3 correspond to a k_z position near $k_z = 0$, close to the k_z plane of the bulk KNLs.

Further, we examine the ARPES photon-energy-dependence of the stoichiometric $InTaS_2$. Two k_x-k_y in-plane Fermi surfaces are shown in Supplementary Fig. 5(a) and Supplementary

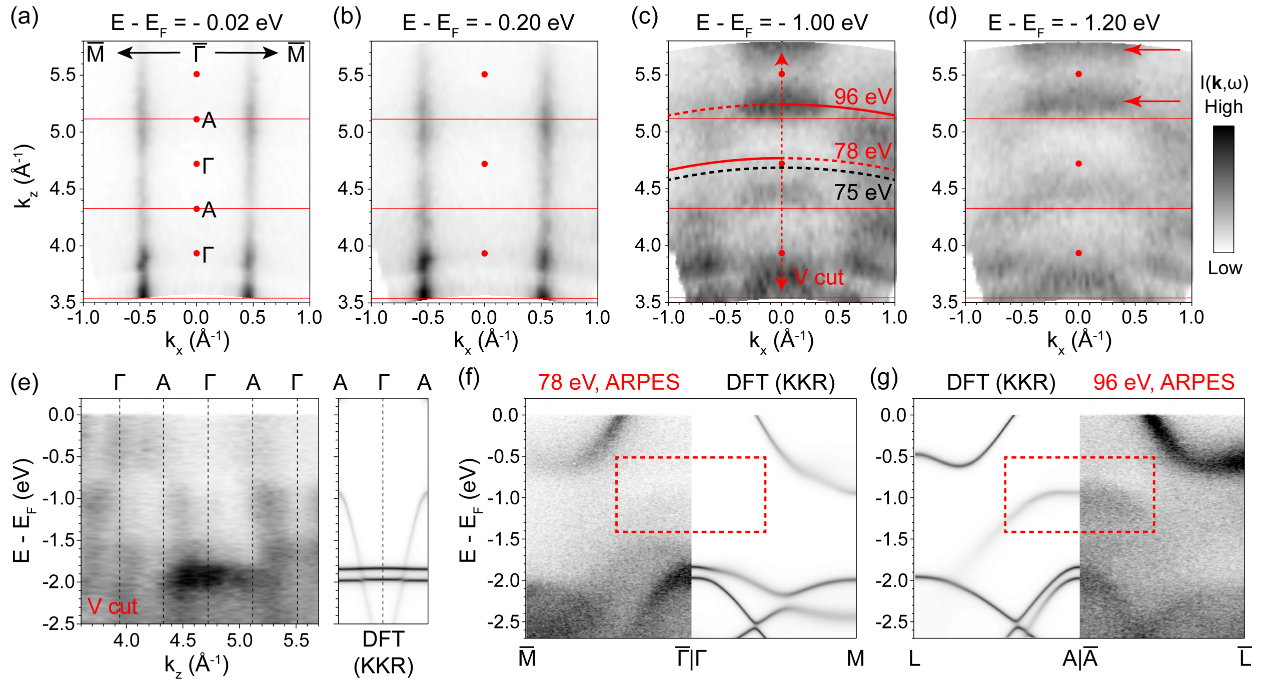

Supplementary Figure 4: Photon-energy-dependent electronic band dispersions of In_xTaS_2 measured by ARPES. (a-d) Photon-energy-dependent k_x-k_z mappings of In_xTaS_2 at binding energies of 0.02, 0.20, 1.00, and 1.20 eV, respectively. k_z corresponds to the varying photon energy and k_x is along the $\bar{\Gamma} - \bar{M}$ high symmetry direction. The two red arcs in (c) correspond to the two photon energies utilized in (f) and (g) for the band dispersions, with the solid half being ARPES data and the dashed half replaced by DFT results at the designated constant k_z. The black dashed curve of 75 eV corresponds to the photon energy used for the data in main text Figs. 2 and 3. (e) $E - k_z$ band dispersions extracted along the "V cut" direction indicated by the vertical red arrow in panel (c). The direction contains $\Gamma - A$, of which the DFT result is provided with on the right. (f) and (g) ARPES band structure along $\Gamma - M$ ($k_z \approx 0$) and $A - L$ ($k_z \approx \pi$) at 78 and 96 eV, respectively, in comparison with the DFT results. The red dashed boxes denote the distinct features at around 1 eV binding energy for the two different photon energies. All data in this figure were acquired around 20 K. An inner potential of 13 eV was experimentally adopted for the $h\nu$ to k_z conversion.

Fig. 5(b). Despite the different photon energies of 74 eV and 62 eV, they show similar band features as calculated in main text Fig. 4(b), but with certain intensity modulation due to the kinetic-energy-dependent matrix element effects. Subsequently, we focus on the $\bar{\Gamma} - \bar{M}$ cut spanned in Supplementary Fig. 5(a) and vary the photon energy in an approximated step of 10 eV, as shown in Supplementary Figs. 5(c1)-(c7). Based on the assumption of a 15 eV inner potential and the c lattice constant of $InTaS_2$, a 10 eV step in our scanned photon energy range would roughly correspond to a change from $k_z = 0$ to $k_z = \pi$. However, as shown in Supplementary Fig. 5(c), we do not observe clear k_z dispersions within the measured binding energy range. We attribute this to the strong k_z broadening effect in $InTaS_2$, which is also one of the motivations for the bulk-sensitive quantum oscillation measurements on $InTaS_2$ presented in main text Fig. 5.

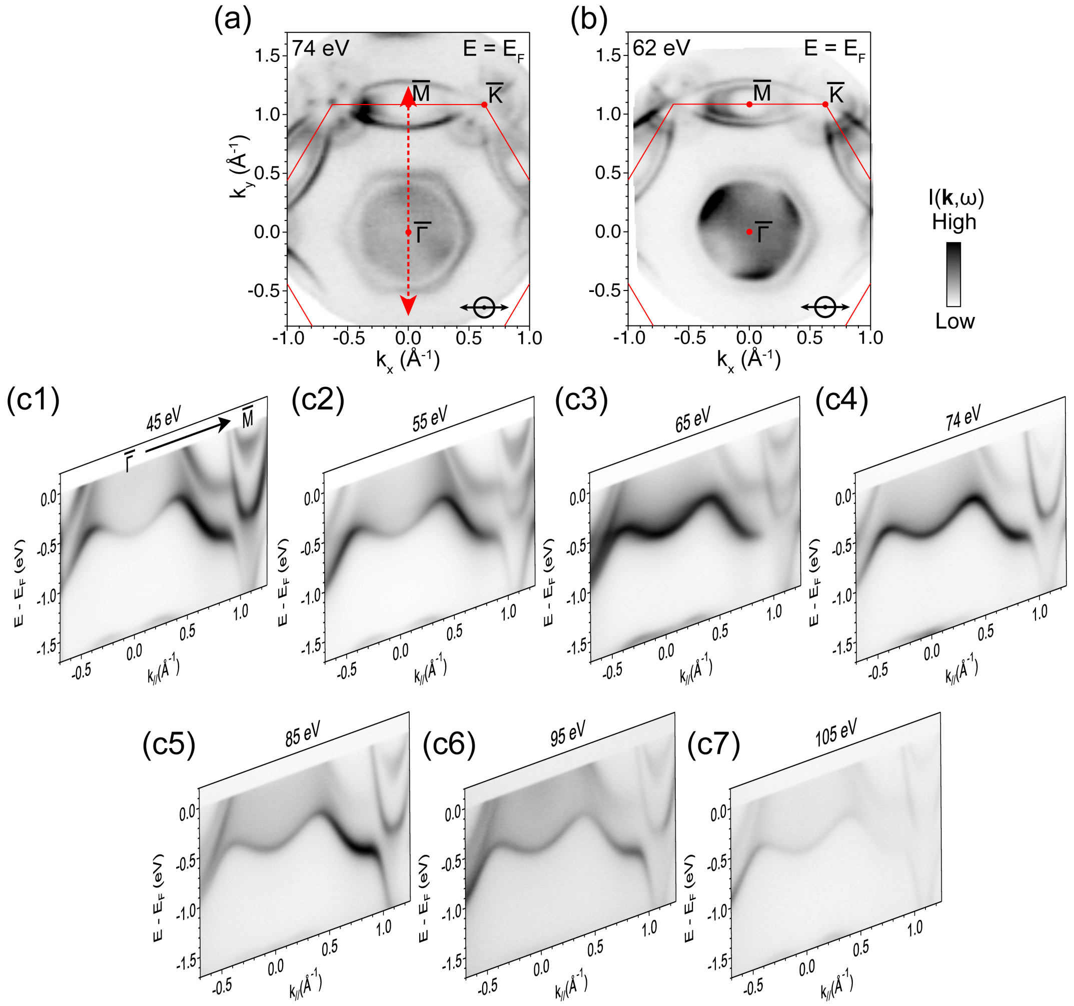

Supplementary Figure 5: Photon-energy-dependent ARPES band structure of $InTaS_2$. (a) and (b) In-plane Fermi surface contours of $InTaS_2$ measured by 74 and 62 eV photons. (c1)-(c7) $\bar{\Gamma} - \bar{M}$ band dispersions obtained with a sequence of increasing photon energies: 45, 55, 65, 74, 85, 95, and 105 eV. The momentum range of (c1)-(c7) is also indicated by the vertical red dashed arrow in panel (a). Light polarization is the same with that of the main text Fig. 4. The measurement temperature is around 23.5 K.

Supplementary Note 5: Angle-resolved photoemission termination dependence of $InTaS_2$ and $In_{1/2}TaS_2$

Since the crystal structure of $InTaS_2$ allows two equally possible cleaving terminations during ARPES measurements, in the main text, we compare the ARPES data with a summation of In and S terminations on the Fermi surface, as reproduced by Supplementary Fig. 6(a) and Supplementary Fig. 6(b1). Furthermore, we decompose the Fermi surface (FS) contributions from the two terminations in Supplementary Fig. 6(b2) and Supplementary Fig. 6(b3). As can be seen from Supplementary Fig. 6(b2), the In termination contributes to the arc-like surface states connecting the $\bar{K}$ and $\bar{K}'$ points across the $\bar{\Gamma} - \bar{M}$ direction, denoted by the red arrow and the "ISS" label. For the S termination, the surface states manifest as a pair of spin-orbital-split hexagonal pockets enclosing the broadened intensities centered around $\bar{\Gamma}$ and the trefoil-like pockets centered around $\bar{K}$ ($\bar{K}'$) points. They are denoted by the two blue arrows and the label "SSS". The termination-dependent band structure along $\bar{\Gamma} - \bar{M}$ is then elaborated in Supplementary Figs. 6(c)-(e), devoted to ARPES experimental data with co-existing In + S terminations, theoretical pure In termination, and theoretical pure S termination, respectively. It is evident again the results in Supplementary Fig. 6(c) can be best described by a summation of the results in Supplementary Figs. 6(d) and (e). The "ISS" in Supplementary Fig. 6(d) gives out the arc-like feature in Supplementary Fig. 6(b2) at the FS, while the "SSS" band in Supplementary Fig. 6(e) manifests as the pinch point along $\bar{\Gamma} - \bar{M}$ on the hexagonal Fermi pockets enclosing $\bar{\Gamma}$ in Supplementary Fig. 6(b3). Notice that the "SSS"-band presented in Supplementary Fig. 6(e) is split from the bulk continuum, echoing the two-peak feature along $\bar{\Gamma} - \bar{M}$ predicted by the one-step model for the S-terminated $In_{1/2}TaS_2$ in Supplementary

Fig. 7(c).

In terms of $In_{1/2}TaS_2$, it is in principle possible to have both In/Vc (Vc: vacancy) and S terminations, similar to the case of $InTaS_2$. However, we do not have unambiguous experimental observation of the S termination and have only obtained high-quality ARPES data on the In/Vc termination, of which the band dispersions along $\bar{\Gamma} - \bar{M} - \bar{K} - \bar{\Gamma}$ are reproduced in Supplementary Fig. 7(a). To quantitatively compare the fine structure near the Fermi level between experiment and theory, we present the momentum distribution curves (MDCs) integrated between E_F and E_F - 60 meV on top in arbitrary unit. The experimental data along $\bar{\Gamma} - \bar{M}$ clearly shows a one-peak structure in Supplementary Fig. 7(a). This is in excellent agreement with the results predicted by the one-step model of photoemission, shown in Supplementary Fig. 7(b), adopting an In/Vc termination. In addition to the consistent one-peak structure along $\bar{\Gamma} - \bar{M}$, the two-peak structure along $\bar{M} - \bar{K}$ in Supplementary Figs. 7(a) and (b) exhibits the same line profile where the right peak is higher than the left peak. These results are in sharp contrast with the one-step model prediction in Supplementary Fig. 7(c) adopting a S termination, where a double peak splitting is observed along $\bar{\Gamma} - \bar{M}$ and along $\bar{M} - \bar{K}$ the right peak is lower than the left peak under the same input of experimental measurement geometry. Therefore, we conclude that our experimentally probed termination is the In/Vc termination. We acknowledge that the matching between Supplementary Fig. 7(a) and Supplementary Fig. 7(b) is not perfect, as the $\bar{K} - \bar{\Gamma}$ MDC line profile cannot be exactly reproduced by either Supplementary Fig. 7(b) or Supplementary Fig. 7(c). Experimentally, it is not straightforward to directly identify the S termination through the double-peak feature along $\bar{\Gamma} - \bar{M}$, since one must carefully distinguish this from band splitting caused by potential cleaving

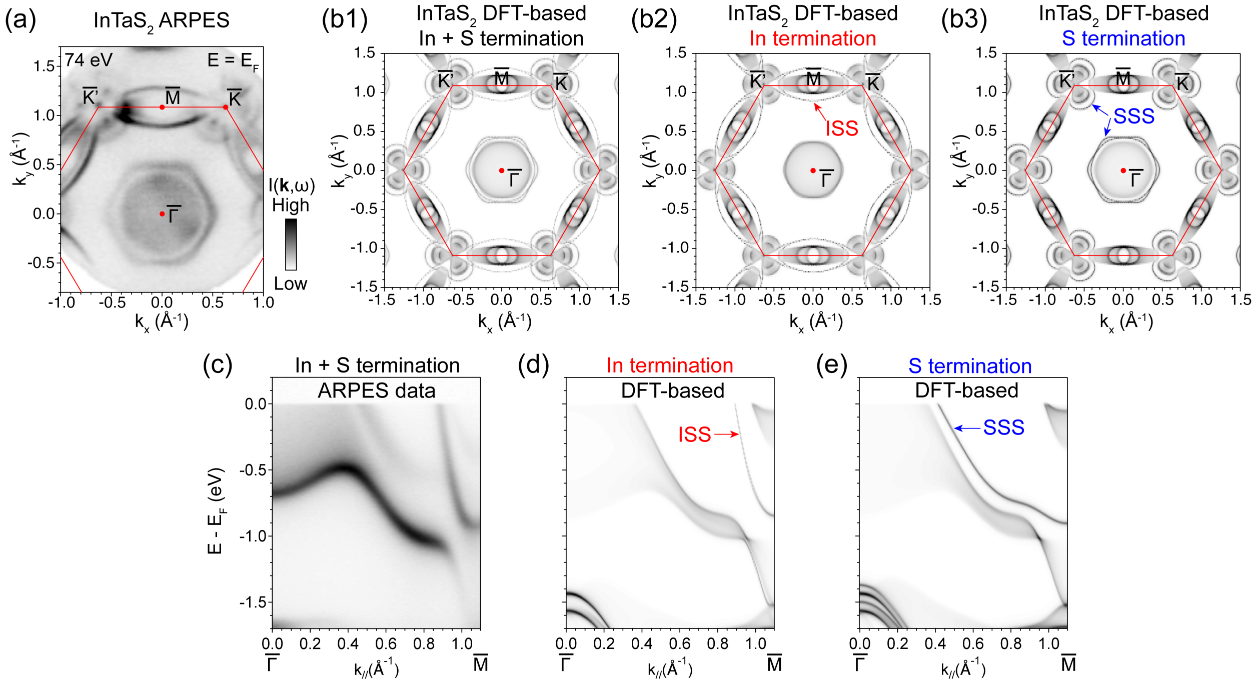

Supplementary Figure 6: Termination-dependent electronic band structure of $InTaS_2$ (a) Fermi surface (FS) measured by 74 eV photons, reproduced from the main text Fig. 4(a). (b1), (b2), (b3) DFT-based (after Wannierization) surface calculations of the FS with summed In and S terminations, In termination, and S termination, respectively. (c) Band dispersions along $\bar{\Gamma} - \bar{M}$ within the same measurement shown in panel (a), with co-existing In and S terminations under the beam spot. (d) DFT-based surface calculations for the $\bar{\Gamma} - \bar{M}$ band structure projected to the In termination. (e) Same as (d) but projected to the S termination. ISS: indium surface states. SSS: sulfur surface states. Calculation results in (d) and (e) are multiplied by a Fermi-Dirac distribution function at 23.5 K convolved with a hypothetical experimental energy resolution of 20 meV at the Fermi level. All calculations presented here have no E_{F} adjustment.

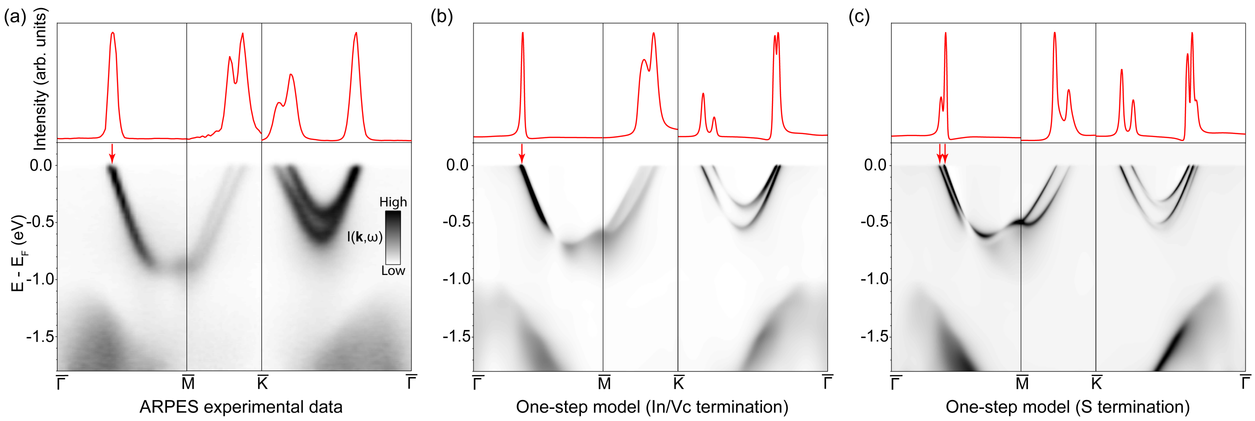

Supplementary Figure 7: Termination-dependent electronic band structure of $\mathrm{In_{1/2}TaS_2}$ (a) Band dispersions measured by 75 eV p-polarized photons along $\bar{\Gamma} - \bar{M} - \bar{K} - \bar{\Gamma}$, reproduced from the main text Fig. 2(f). All red curves on top are the momentum distributions curves (MDCs) integrated from E_{F} to E_{F} - 60 meV along the corresponding high symmetry momentum directions indicated at the bottom. (b) One-step ARPES calculations using the In/Vc termination, reproduced from main text Fig. 2(g), but with the integrated MDCs attached on top. Vc: vacancy. (c) One-step ARPES calculations using the same experimental conditions but with the S termination instead. The vertical red arrows between the MDCs and the band dispersion images are indicators for the one-peak or two-peak structure. All presented one-step model calculated data have a Fermi level adjustment of +75 meV, consistent with the operation in main text Fig. 2, and are applied with a Fermi-Dirac distribution function at 15 K, which is additionally convolved with a hypothetical experimental energy resolution of 20 meV at the Fermi level.

domains, because the material does not usually give a large area of single cleaving domain. Future experiments with smaller beam spot, combined with sulfur core-level spectroscopy study can further clarify and confirm the S termination scenario of $In_{1/2}TaS_2$.

Supplementary Note 6: Kramers nodal lines in the isostructural $PbTaSe_2$

To demonstrate the generic presence of KNLs in the $P\bar{6}m2$ intercalated transition metal dichalcogenide (TMD) material family, we present the angle-resolved photoemission spectroscopy (ARPES) observation of the KNLs in $PbTaSe_2$ which is isostructural to $InTaS_2$ and has a debated nature of its superconductivity onset around 3.7 K [3–7]. In addition, previous study has identified Weyl nodal rings centered around H point from first-principles calculations [8]. However, the bulk nodal lines above the Fermi level there hinder a direct observation in ARPES. KNLs arising from the achiral little group symmetry in $PbTaSe_2$ are fundamentally distinct from the previously proposed Weyl nodal lines.

As shown in Supplementary Fig. 8(a), the constant energy contour at E - E_{F} = -0.1 eV best showcases the presence of the Γ-M KNLs indicated by the six purple lines. The direct consequence is that all pockets intersected by the KNLs are enforced to be Kramers degenerate. This can be directly seen from the pair of hexagonal pockets centered around $\bar{\Gamma}$, of which the pinch points are marked by the open black circles along the six Γ-M directions. To visualize the band dispersions of the KNL enforced pinch points, we take two momentum paths indicated by the blue and red dashed arrows in Supplementary Fig. 8(a), intersecting the Γ-M KNLs twice and once, respectively.

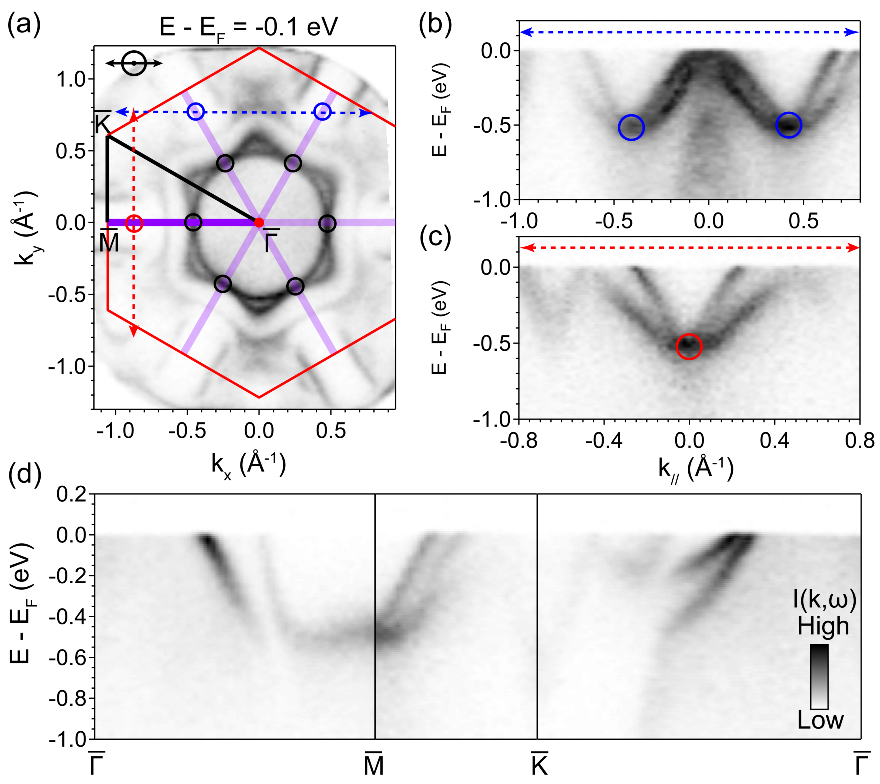

Supplementary Figure 8: ARPES observation of Kramers nodal lines and their pinch points in $PbTaSe_2$. (a) Measured constant energy contour of $PbTaSe_2$ at 0.1 eV binding energy. (b) Band dispersions corresponding to the blue dashed arrow in (a). The pinch points are denoted by open blue circles in both (a) and (b). (c) Same as (b) but for the red vertical dashed arrow in (a). The pinch points are denoted by the open red circles in (a) and (c). (d) Electronic band structure along $\bar{\Gamma}$-$\bar{M}$-$\bar{K}$-$\bar{\Gamma}$, where the path is outlined by the thick purple line and solid black lines in (a). All of the data in the figure were measured by 77 eV and linear horizontal polarized photons of which the direction is indicated on the top left of (a). The measuring temperature at the sample is estimated to be around 25 K.

As a result, their band dispersions show respectively two and one pinch point in Supplementary Fig. 8(b) and (c), as highlighted by the blue and red open circles. Lastly in Supplementary Fig. 8(d), the electronic band structure along $\bar{\Gamma}$-$\bar{M}$-$\bar{K}$-$\bar{\Gamma}$ is displayed, in a similar fashion to the main text Fig. 4(c). It is straightforward to see that the bands along $\bar{\Gamma}$-$\bar{M}$ are the two-fold degenerate KNLs and split into two spin-orbital branches along $\bar{M}$-$\bar{K}$-$\bar{\Gamma}$. Therefore, all of the experimental evidence above points to the robust existence of the KNLs in $PbTaSe_2$ and their critical role in determining the Fermi surface topology.

Supplementary Note 7: First-principles calculations and quantum oscillations on $InTaS_2$

In order to build correspondence between Fermi surfaces of $InTaS_2$ and quantum oscillation (QO) frequencies in main text Figs. 4(d-i), we compared the QO frequencies from experiments to the value of extremal cross-sectional area from density functional theory (DFT) calculations. In the original calculation (Supplementary Fig. 9(a)), the size of the β cross-sectional area is 221 T, which is close to the experimental value (213 T); the size of the ϵ cross-sectional area is 4689 T, which is smaller than the experimental value (6000 T).

The QOs of 6000 T and 12000 T might be a consequence of the magnetic breakdown effect or magnetic interaction effect [9], where $F_\epsilon = F_1 + F_2$. If that is the case, since $F_{2\epsilon}$ can be identified in the FFT spectrum, the peaks corresponding to QOs of frequency $F_1 + 2 * F_2$ and $2 * F_1 + F_2$ of similar amplitude are expected in the FFT spectrum as well. However, as shown in Supplementary

Fig. 10(b), no FFT peaks of similar amplitude are found between 6000 T and 12000 T. This implies that the QOs of frequency 6000T and 12000 T are fundamental oscillations and the second harmonics of one Fermi surface cross-sectional area.

The ϵ cross-sectional area has the largest cross-sectional area in the Brillouin zone, and if the underlying energy bands (red and green in Supplementary Fig. 9(a)) are shifted up by 128 meV, ϵ cross-sectional area will become 5997 T, which matches the experimental data (Supplementary Fig. 9(b). Such band shifting will create an additional cross-sectional area α with a cross-sectional area of 158 T simultaneously. The size of this cross-sectional area is close to the experimental value of F_{α}=104 T. The value of the cross-sectional area α between experiments and theory can be matched if the underlying energy bands (red and green in Supplementary Fig. 9(a)) are shifted up by 95 meV. A great match between experimental ARPES results and DFT results is achieved when a similar band shifting is adopted.

In addition to the frequencies from α, β, and ϵ, the QOs of $InTaS_2$ consists of other frequencies, as can be seen in the inset of Supplementary Fig. 10(a). This is also confirmed in the FFT spectrum (Supplementary Fig. 10(b)), where several peaks with frequencies higher than $2F_{\beta}$ can be found in the spectrum. This is consistent with the complex Fermi surface from DFT calculations.

Figures 11(a-b) show the Shubnikov de-Haas (SdH) oscillations of $InTaS_2$ at different magnetic field orientations up to 44.8 T at 0.35 K. The measurement configuration is the same as the sketch in the inset of the main text Fig. 4(g). The QOs from the α and β pockets can be seen as the large wiggles in Supplementary Fig. 11(a). The quantum oscillations that are distinct from α

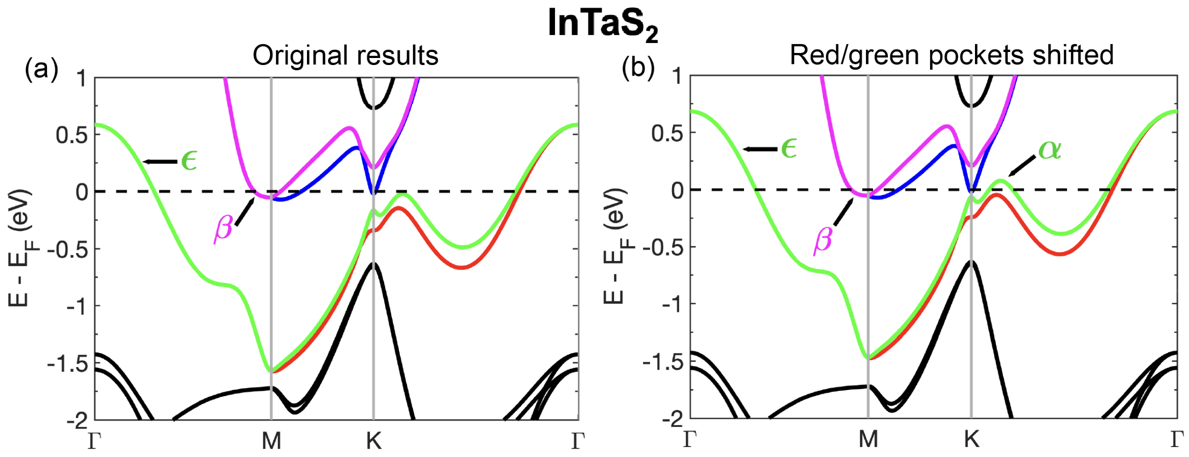

Supplementary Figure 9: Band structure of $InTaS_2$ along Γ-M-K-Γ paths. The colors of the Fermi surfaces are kept the same with the main text Fig. 1(e). (a) Band structure from original DFT calculation (without shifting the bands). (b) Band structure used to match quantum oscillation (after shifting the bands). The red and green bands are shifted up by 128 meV, and the magenta and blue bands are shifted up by 3 meV.

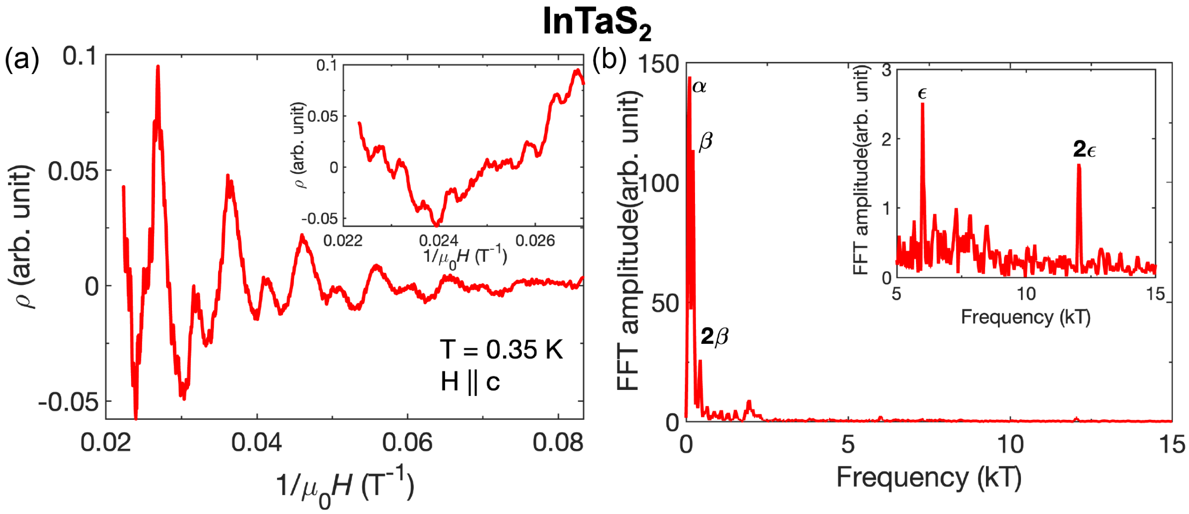

Supplementary Figure 10: Shubnikov de-Haas (SdH) oscillations of $InTaS_2$ at 0.35 K with H$\|$c. (a) SdH oscillations of $InTaS_2$ after a smooth background subtraction. The inset is a zoom-in view of the oscillations above 37 T, which shows oscillations distinct from α, β, and ϵ. (b) FFT spectrum of (a). The inset is a zoom-in view of the spectrum between 5000 T and 15000 T.

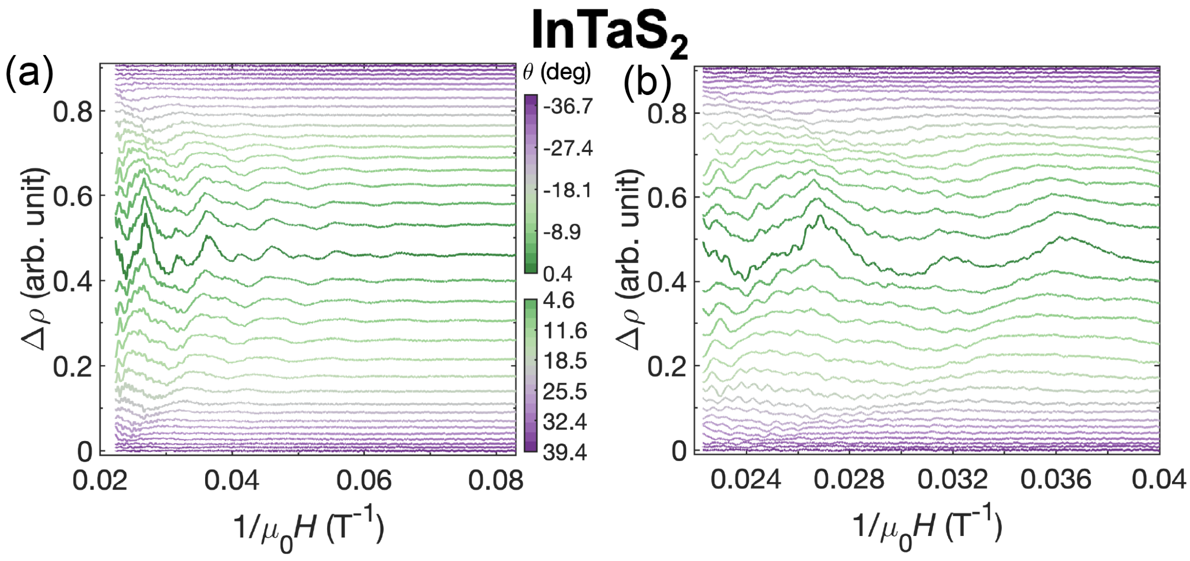

Supplementary Figure 11: Shubnikov de-Haas (SdH) oscillations of $InTaS_2$ with different field orientations up to 44.8 T at 0.35 K. The measurement geometry is the same as the schematic in the inset of main text Fig. 4(g). (a) SdH oscillations of $InTaS_2$ after a smooth background subtraction from 12 T to 44.8 T with different magnetic field orientations. (b) Zoom-in view of (a) from 25 T to 44.8 T, illustrating quantum oscillations of frequencies distinct from α, β, and ϵ.

and β can be seen as the smaller wiggles in Supplementary Figs. 11(a,b). Similar to α and β, the amplitude of these oscillations becomes too weak to be observed when the field is tilted away from the c-axis.

Supplementary Note 8: Chiral Majorana modes and anomalous Hall effect from an ideal Kramers nodal line

In 3D topological insulators, the massless 2D Dirac node on the surface has a number of fascinating consequences that are forbidden in strictly 2D systems. When gapped out by superconductivity, a vortex in the surface superconductor traps a single Majorana zero mode. If, instead, the Dirac node is gapped out by magnetization or Zeeman mass M, it leads to a strange half-integer anomalous Hall conductivity $\sigma_{\text{Hall}} = -\text{sgn}(M)e^2/2h$. Both effects are rooted in the topological nature of the system and can be viewed as signatures of a π-flux, Berry curvature singularity at the origin of a massless Dirac node. Inspired by the discovery of Kramers nodal lines (KNLs) in InTaS_2, we describe analogous behaviors stemming from general KNLs. Similar to the Dirac node on the topological insulator surface, the underlying principle is that the KNLs are singularities of Berry curvature. However, fundamental differences in dimensionality and geometry produce richer behaviors in the latter.

A single Kramers nodal line

For simplicity, we assume a hypothetical continuum band structure with a single, isotropic Kramers nodal line (KNL) along k_z. Anisotropies are unimportant for our main results as they rely on the topological Berry flux carried by the KNL. A generic two-band Bloch Hamiltonian for a KNL is of the form:

$$H_0(\mathbf{k}) = \begin{pmatrix} \beta_{k_z} k_+^m + \beta_{k_z}^* k_-^m & \alpha_{k_z} k_-^n \\ \alpha_{k_z}^* k_+^n & -\beta_{k_z} k_+^m - \beta_{k_z}^* k_-^m \end{pmatrix} + \epsilon(\mathbf{k}) \tag{1}$$

$$\equiv \mathbf{d}(\mathbf{k}) \cdot \boldsymbol{\sigma} + \epsilon(\mathbf{k}) \tag{2}$$

where $\boldsymbol{\sigma}$ is a vector of Pauli matrices in spin space, $k_\pm = k_x \pm ik_y$, $m, n \in \mathbb{Z} \geq 0$ and $\epsilon(\mathbf{k})$ is a smooth function that preserves all the symmetries of the spin-dependent part of $H_0(\mathbf{k})$. Fermi surfaces produced by $H_0(\mathbf{k})$ are given by $|\mathbf{d}(\mathbf{k})| = \pm\epsilon(\mathbf{k})$. They touch on the k_z axis when $\epsilon(\mathbf{k}) = 0$, forming the KNL. For simplicity, we assume $\epsilon(0, 0, k_z) = 0$ has precisely two solutions $k = \pm K_z$. Away from the KNL, the Fermi surfaces form a "spindle" if $\epsilon(\mathbf{k}) = 0$ is a closed surface enclosing $k_z = 0$ or π, or an "octdong" if $\epsilon(\mathbf{k}) = 0$ is a set of disconnected open surfaces that extend out to $|k_\pm| \to \infty$. Supplementary Figures 12(a,b) show a schematic of a single KNL along the k_z axis and a corresponding spindle Fermi surface.

The Berry flux carried by the KNL can be read off from the the form of $H_0(\mathbf{k})$. If $m < n$ and $\beta_{k_z} \neq 0$, or if $\alpha_{k_z} = 0$, $H_0(\mathbf{k})$ is dominated by the diagonal terms for small $k_{x,y}$, causing spin to be polarized along $\pm\hat{z}$ near the KNL and ensuring the Berry flux carried by it to vanish. In the complementary regime, namely, $\alpha_{k_z} \neq 0$ and either $m > n$ or $\beta_{k_z} = 0$, the spin direction winds n times in the xy plane around the KNL and yields a Berry phase of $\pm n\pi$.

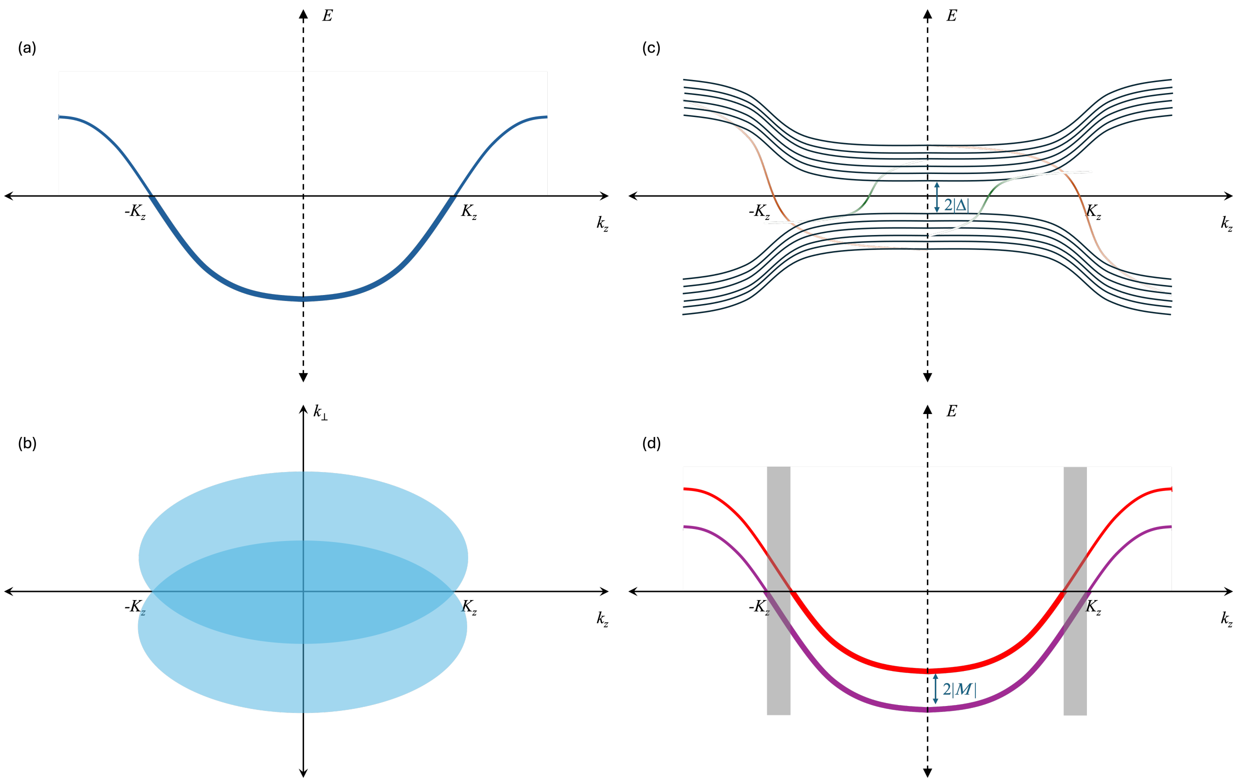

Supplementary Figure 12: Vortex modes and anomalous Hall effect from a single KNL. Schematic of (a) a single KNL along k_z with a thick (thin) line denoting occupied (empty) states, and (b) the corresponding spindle Fermi surface consisting of two Fermi surfaces touching at two points, $(0, 0, \pm K_z)$. Here, $k_\perp = \sqrt{k_x^2 + k_y^2}$. (c) Superconductor vortex modes when the Berry phase around the KNL is an odd multiple of π. Orange modes stem from the pinch points at $\pm K_z$ while green modes stem from cross-sections of the outer Fermi surface around which the Berry phase is $\pi(\mod 2\pi)$. When the Berry phase is an even multiple of π, no protected in-gap modes exist. (d) Origin of the anomalous Hall conductivity with thick (thin) lines indicating filled (empty) states. When an exchange or Zeeman field M splits the KNL, anomalous Hall conductivities from the two bands cancels for most k_z slices except those shaded grey, which leads to a net σ_{xy}. The qualitative nature of the vortex modes and anomalous Hall conductivity is independent of whether the normal state has a spindle or a octdong Fermi surface.

Chiral Majorana modes

Shortly after Fu and Kane's seminal result [10], the role of the Fermi surface Berry phase in the existence of the zero mode was uncovered [11]. In particular, it was pointed out that a zero mode exists in the vortex spectrum if and only if the normal state 2D Fermi surface (or 2D cross-section of a 3D Fermi surface) has a Berry phase of π modulo 2π. Although the results of Fu and Kane [10], and Hosur et al. [11] focused on complementary parameter regimes defined by point-like and large Fermi surfaces, respectively, and yield distinct overall spectra for in-gap vortex modes, they concur on the existence of the zero mode. In addition, the connection between Berry phases and vortex spectrum was found to also hold for semiclassical orbits consisting of Fermi arcs and bulk chiral modes in Weyl semimetals, where the notion of a large, smooth Fermi surface is ill-defined [12]. Thanks to these observations, we anticipate the connection between 2D Fermi surface Berry phases and superconductor vortex spectra – especially the zero modes – to hold broadly. For KNLs, we expect those with odd n and $m > n$ to produce vortex zero modes when they intersect the 3D Fermi surface.

To prove this explicitly, we consider a unit superconductor vortex $\Delta(\mathbf{r}) = |\Delta(r)|\, e^{i\theta}$ along k_z, where (r, θ, z) define a cylindrical co-ordinate system in real space. We focus on $k_z = K_z$, where the KNL is at the Fermi level. We also ignore the diagonal terms in $H_0(\mathbf{k})$; this is exact if $\beta_{k_z} = 0$ or justifiable *post hoc* if the superconductor coherence length ξ is large and $m > n$. These assumptions yield the following Bogoliubov-deGennes Hamiltonian in the ba-

sis $\left(c_\uparrow(\mathbf{r}, K_z), c_\downarrow(\mathbf{r}, K_z), c^\dagger_\downarrow(\mathbf{r}, K_z), -c^\dagger_\uparrow(\mathbf{r}, K_z)\right)^T$:

$$H_{\text{BdG}}[\Delta] = \begin{pmatrix} & \alpha_{K_z} k_-^n & |\Delta(r)|\, e^{i\theta} & \\ \alpha^*_{K_z} k_+^n & & & |\Delta(r)|\, e^{i\theta} \\ |\Delta(r)|\, e^{-i\theta} & & & -\alpha_{K_z} k_-^n \\ & |\Delta(r)|\, e^{-i\theta} & -\alpha^*_{K_z} k_+^n & \end{pmatrix} \tag{3}$$

Working in polar co-ordinates, it is easy to verify that the following is a normalizable solution to $H_{\text{BdG}}\psi = 0$ when n is odd:

$$\psi(r,\theta) = \begin{pmatrix} \alpha_{K_z} e^{-i(n-1)\theta/2} \\ 0 \\ 0 \\ \alpha^*_{K_z} e^{i(n-1)\theta/2} \end{pmatrix} i^n r^{-\frac{n-1}{2}} \exp\left[-\frac{1}{|\alpha_{K_z}|^2} \int_0^r dr_1 \int_0^{r_1} dr_2 \ldots \int_0^{r_{n-1}} dr_n\, |\Delta(r_n)|\right] \tag{4}$$

For $n = 1$, $\psi(r,\theta)$ reduces to the familiar Majorana zero mode found by Fu and Kane [10]. We have shown that similar Majorana zero modes exist for all odd n. On the other hand, we could not find zero modes for even n. While this does not prove their absence, they are not expected based on general Berry phase arguments mentioned above and derived in Ref. [11].

Away from $k_z = K_z$, the normal state dispersion quickly dominates the pairing – $|\epsilon(0,0,k_z)| \gg |\Delta(r)|$ – and the eigenstates of H_{BdG} are almost purely electron-like or hole-like. The zero mode, in particular, mixes with all the non-chiral states in the vortex and yields an overall chiral mode that essentially tracks either $\epsilon(0,0,k_z)$ or $-\epsilon(0,0,k_z)$. The choice of sign is such that as k_z is varied, it preserves the homotopy $\pi_3(S_2) = \mathbb{Z}$ of the mapping from (x, y, k_x, k_y) compactified to S_3 to the direction of the effective spin-1 constructed from the physical spin-1/2 and Nambu spin-1/2.

The details of this mapping will be discussed elsewhere. The current work focuses merely on the existence of the zero mode at K_z and its nontrivial chirality away from K_z.

If the Fermi surfaces form a spindle, there is necessarily a Fermi surface cross-section at finite (k_x, k_y) and generically a different k_z that carries a Berry phase of $-n\pi$. This ensures that Bloch states are properly regularized in the normal state. In the superconductor vortex, it gives rise to another zero mode when n is odd. If the Fermi surfaces form an octdong, a second zero mode once again appears for odd n because octdong Fermi surfaces themselves appear in pairs to ensure periodicity of the Brillouin zone. In either case, a second chiral mode appears to compensate for the one from the pinch point. The final result is depicted in Supplementary Fig. 12(c).

Anomalous Hall effect

In this section, we consider the effect of a Zeeman or exchange field along the KNL

$$H_m = M\sigma_z \tag{5}$$

The KNL now splits into a pair of modes dispersing as $\pm M + \epsilon(0, 0, k_z)$ along k_z and leads to novel anomalous Hall physics. In particular, for small M, the $n\pi$ Berry flux carried by the KNL gets distributed over a small region around the k_z axis with opposite signs for the two bands. For most k_z values, both bands are either occupied or unoccupied, resulting in zero net Chern number. However, for $-|M| < \epsilon(0, 0, k_z) < |M|$, precisely one of the two modes stemming from the KNL is occupied at zero temperature, and each constant k_z slice in this region yields a Hall conductivity

$-n\text{sgn}(M)e^2/2h$. Integrating over k_z gives the total 3D Hall conductivity per unit length:

$$\frac{\sigma_{xy}}{L_z} \approx -\frac{2ne^2}{h^2}\frac{M}{|v_{\text{KNL}}|} + O(M^2) \tag{6}$$

where $v_{\text{KNL}} = \frac{1}{\hbar}\left.\frac{\partial\epsilon(0,0,k_z)}{\partial k_z}\right|_{k_z=\pm K_z}$ denotes the slope of the KNL at the Fermi level and L_z is the thickness in the z direction. Unlike the vortex zero modes whose existence was tied to the parity of n, the anomalous Hall effect appears for any n. The result is sketched in Supplementary Fig. 12(d). We have assumed that the KNL crosses the Fermi level at precisely two points $k_z = \pm K_z$. If additional pairs of intersections between the KNL and the Fermi level exist, analogous contribution from each pair must be added to σ_{xy} above.

Supplementary References

1. Lian, C. -S, *et al.*, Intrinsic and doping-enhanced superconductivity in monlayer $1H$-TaS_2: critical role of charge ordering and spin-orbit coupling. *Phys. Rev. B.* **105**, L180505 (2022). URL https://journals.aps.org/prb/abstract/10.1103/PhysRevB.105.L180505.

2. Sobota, J. A., He, Y., and Shen, Z.-X., Angle-resolved photoemission studies of quantum materials. *Rev. Mod. Phys.* **93**, 025006 (2021). URL https://journals.aps.org/rmp/abstract/10.1103/RevModPhys.93.025006.

3. Guan, S. -Y., *et al.*, Superconducting topological surface states in the noncentrosymmetric bulk superconductor $PbTaSe_2$. *Sci. Adv.* **2**, e1600894 (2016). URL https://www.science.org/doi/10.1126/sciadv.1600894.

4. Pang, G. M., *et al.*, Nodeless superconductivity in noncentrosymmetric $PbTaSe_2$ single crystals. *Phys. Rev. B.* **93**, 060506(R) (2016). URL https://journals.aps.org/prb/abstract/10.1103/PhysRevB.93.060506.

5. Wilson, M. N., *et al.*, μSR study of the noncentrosymmetric superconductor $PbTaSe_2$. *Phys. Rev. B.* **95**, 224506 (2017). URL https://journals.aps.org/prb/abstract/10.1103/PhysRevB.95.224506.

6. Le, T., *et al.*, Evidence for nematic superconductivity of topological surface states in $PbTaSe_2$. *Sci. Bull.* **65**, 1349-1355 (2020). URL https://doi.org/10.1016/j.scib.2020.

04.039.

7. Zhu, Z. H., *et al.*, Muon spin rotation and relaxation study on topological noncentrosymmetric superconductor $PbTaSe_2$. *New J. Phys.* **24**, 023002 (2022). URL https://iopscience.iop.org/article/10.1088/1367-2630/ac48ea.

8. Bian, G., *et al.*, Topological nodal-line fermions in spin-orbit metal $PbTaSe_2$. *Nat. Commun.* **7**, 10556 (2016). URL https://www.nature.com/articles/ncomms10556.

9. Shoenberg, D., *Magnetic Oscillations in Metals* (Cambridge University Press, Cambridge, England, 1984).

10. Fu, L. & Kane, C. L., Superconducting Proximity Effect and Majorana Fermions at the Surface of a Topological Insulator. *Phys. Rev. Lett.* **100**, 096407 (2008). URL https://journals.aps.org/prl/abstract/10.1103/PhysRevLett.100.096407.

11. Hosur, P. *et al.*, Majorana Modes at the Ends of Superconductor Vortices in Doped Topological Insulators. *Phys. Rev. Lett.* **107**, 097001 (2011). URL https://journals.aps.org/prl/abstract/10.1103/PhysRevLett.107.097001.

12. Giwa, R. & Hosur, P., Superconductor Vortex Spectrum Including Fermi Arc States in Time-Reversal Symmetric Weyl Semimetals. *Phys. Rev. Lett.* **130**, 156402 (2023). URL https://journals.aps.org/prl/abstract/10.1103/PhysRevLett.107.097001.
